# Calcium/Calcineurin Synergizes with Prostratin to Promote NF-κB Dependent Activation of Latent HIV

**DOI:** 10.1371/journal.pone.0077749

**Published:** 2013-10-30

**Authors:** Jonathan K. Chan, Darshana Bhattacharyya, Kara G. Lassen, Debbie Ruelas, Warner C. Greene

**Affiliations:** 1 Gladstone Institute of Virology and Immunology, San Francisco, California, United States of America; 2 Department of Medicine, University of California San Francisco, San Francisco, California, United States of America; 3 Department of Microbiology and Immunology, University of California San Francisco, San Francisco, California, United States of America; George Mason University, United States of America

## Abstract

Attempts to eradicate HIV have been thwarted by the persistence of a small pool of quiescent memory CD4 T cells that harbor a transcriptionally silent, integrated form of the virus that can produce infectious virions following an anamnestic immune response. Transcription factors downstream of T-cell receptor activation, such as NF-κB/Rel and nuclear factor of activated T cells (NFAT) transcription members, are considered important regulators of HIV transcription during acute HIV infection. We now report studies exploring their precise role as antagonists of HIV latency using cell and primary CD4 T cell models of HIV-1 latency. Surprisingly, RNA interference studies performed in J-Lat CD4 T cells suggested that none of the NFATs, including NFATc1, NFATc2, NFATc3, and NFAT5, played a key role in the reactivation of latent HIV. However, cyclosporin A markedly inhibited the reactivation response. These results were reconciled when calcium signaling through calcineurin was shown to potentiate prostratin induced activation of NF-κB that in turn stimulated the latent HIV long terminal repeat (LTR). Similar effects of calcineurin were confirmed in a primary CD4 T cell model of HIV latency. These findings highlight an important role for calcineurin in NF-κB-dependent induction of latent HIV transcription. Innovative approaches exploiting the synergistic actions of calcineurin and prostratin in the absence of generalized T-cell activation merit exploration as a means to attack the latent viral reservoir.

## Introduction

In HIV-infected patients, highly active antiretroviral therapy (HAART) effectively reduces viral loads but cannot eradicate the virus. Instead, the infection persists for decades due to latent virus residing at least in a small pool of CD4 memory T cells (10^6^–10^7^/patient). Despite HAART, viral persistence and low-level HIV replication eventually compromise the immune system and lead to AIDS. New strategies to purge the latent reservoir are urgently needed.

One promising approach involves “flushing” the latent virus from its cellular reservoir while continuing HAART. However, attempts to stimulate latent provirus expression with anti-CD3 or interleukin (IL)-2 were unsuccessful [1], [2]. These disappointing results reflect in part our incomplete understanding of how latent HIV-1 transcription is induced during activation of infected resting CD4 memory cells *in vivo*. Combining prostratin, a non-tumorigenic phorbol ester, and histone deacetylase (HDAC) inhibitors have been suggested as an effective way to purge the latent reservoir [3]. Reactive oxygen species activators that stimulate latent HIV transcription without the undesirable effects of global T-cell activation have also been described [4], [5]. Of note, both of these strategies involve the induction of NF-κB.

Mounting evidence suggests that members of the NF-κB transcription factor family (RelA, RelB, c-Rel, p50/p105, and p52/p100) regulate HIV transcript production [6]–[12]. The enhancer region, located –104 to –80 from the transcriptional start site of the 5′ long terminal repeat, has two identical NF-κB binding sites that are highly conserved among HIV-1 subtypes [13]. Knockdown of RelA severely reduces HIV transcription during acute infection [14]–[16]. Using cellular models of HIV latency such as J-Lat cells, we showed that, in the absence of cellular stimulation, NF-κB p50 homodimers occupy the LTR-κB sites of latent HIV and recruit HDAC1 to promote a condensed chromatin state through histone hypoacetylation, which reduces the binding of RNA polymerase II. Conversely, upon stimulation of cell-surface receptors, such as T-cell receptor (TCR) or Tumor Necrosis Factor (TNF)-α receptor, or with phorbol esters, such as prostratin, RelA/p50 complexes translocate to the nucleus and engage the duplicate κB sites present in the LTR. In this process they displace bound p50 homodimers and recruit PTEF-b. PTEF-b in turn phosphorylates serine 2 within the carboxyl heptad repeats of RNA polymerase II, enhancing its transcriptional elongation [6], [12].

Several nuclear factors of activated T cells (NFAT) transcription factors also appear to regulate HIV-1 transcription during acute infection. For example, NFATc1, NFATc2, and NFAT5 engage the HIV-LTR with similar κB site preference as RelA/p50 [17]–[20]. Moreover, ectopic expression of NFATc1 and NFATc2 induces LTR-luciferase reporter activity and increases HIV infectivity in T cells [17], [19], whereas knockdown of NFAT5 decreases viral infection in macrophages [20]. However, much less is known on the role of NFAT family members as antagonists of HIV latency.

Recently, various strategies have been used to generate primary CD4 T cells latently infected with HIV [5], [13], [21]–[24]. In many of these models, reactivation of latent HIV by TCR crosslinking is reduced by cyclosporin A (CsA), which inhibits calcineurin [5], [21]. Since the NFATs are the best-known targets of calcineurin, NFAT has been suggested to play a role in driving HIV out of its latent state. However, a number of studies have also argued for an NFAT-independent function of calcineurin in TCR-induced activation of NF-κB in T cells [25]–[29]. Therefore, it is unclear whether the observed CsA-sensitivity in latent HIV transcription in these primary T-cell models is a function of calcineurin-induced NFAT activity at HIV-LTR, or whether it reflects calcineurin modulating the NF-κB pathway or perhaps both.

In this study, we employed a combination of *in vitro* and primary T-cell models of HIV latency to demonstrate that the NFATs are unlikely to be the predominant factors driving HIV out from latency. Rather, our findings reinforce the notion that RelA is an important antagonist of HIV latency and that maximal NF-κB induction involves the action of calcineurin after T-cell activation.

## Methods

### Ethics Statement

This study was conducted according to the principles expressed in the Declaration of Helsinki. All individuals provided written informed consent for the collection of samples and subsequent analysis as approved by the Institutional Review Board of Stanford University Blood Bank.

### Cell Lines and Cell Culture Conditions

Jurkat cells (obtained from American Type Culture Collection) and TCR-J-Lat clone 5A8 were cultured in RPMI 1640 supplemented with 10% fetal bovine serum, penicillin, streptomycin, and L-glutamine. Cells were stimulated with phorbol-12-myristate-13-acetate (Calbiochem) or prostratin (Sigma) at various dosages, as indicated, in the presence or absence of 2 µM ionomycin (Sigma). Cells were also stimulated with 10 ng/ml TNF-α(R&D Systems) or 10 µg/ml anti-CD3 (clone OKT3) antibodies bound to 24-well plates (Calbiochem) with 1 µg/ml soluble anti-CD28 antibodies (BD Pharmigen) at the times indicated. To inhibit calcineurin, cells were pretreated with 500 nM CsA (Sigma Aldrich) for 2 h before stimulation.

### Latently Infected TCR-J-Lat Clones

To generate latently infected J-Lat clones, Jurkat cells were infected with VSV-G pseudotyped HIV-R7/*env*−/GFP [30] at a multiplicity of infection of 0.1 for 96 h. This single-round full-length HIV molecular clone contains a GFP reporter gene in place of *nef* and a frameshift mutation in *env*. GFP-positive cells were sorted twice with a FACSDiva cell sorter (Becton Dickinson) and discarded. GFP-negative cells were propagated for 1 week and stimulated with plate-bound anti-CD3 and soluble anti-CD28 antibodies for 12 h. GFP-positive cells were collected and propagated without stimulation to allow silencing of LTR transcription. Single cells were seeded in 96-well plates to generate cell clones. Six clones in which LTR activity was silenced, as shown by repetitive Alu-gag PCR [31], had the same integration site. One of these clones, 5A8, was used in all subsequent experiments.

### NFAT-DsRed2 or κB-DsRed2 Reporter Cells

To generate κB-DsRed2 and NFAT-DsRed2 reporter cells, we transduced 5A8 cells with lentiviral vectors PPT-κB-DsRed2 and PPT-NFAT-DsRed2, respectively, for 72 h [32]. DsRed2-negative cells were isolated by fluorescence-activated cell sorting (FACS) and treated with 10 µg/ml plate-bound anti-CD3 (clone OKT3) and 1 µg/ml soluble anti-CD28 antibodies for 24 h. Cells with TCR-inducible DsRed2 expression were selected by flow cytometry and maintained.

### Stimulation Experiments Involving NF-κB and NFAT Knockdown

siRNAs were constructed by Ambion as follows: 5′-GGACAUAUGAGACCUUCAAtt-3 (RelA siRNA); 5′-ACACCAAAGUCCUGGAGAUtt-3 (both NFATc1 and NFATc2 siRNA); 5′GGACAUCUCUUAGCCCAUAtt-3′ (NFATc3 siRNA) and 5′-CAACAUGC CUGGAAUUCAA-3′ (NFAT5 siRNA). Silencer negative control #1 siRNA (AM4611) was used as a negative control or to normalize the total amount of nucleofected siRNA at 300 pmol across all conditions. siRNA was introduced twice by Amaxa nucleofection for 2 days. On day 3, cells in each siRNA condition were divided into two fractions, treated with dimethyl sulfoxide (DMSO) or 500 nM CsA for 2 h, and stimulated with plate-bound anti-CD3 and soluble anti-CD28 for 24 h. Cells were collected and analyzed with a FACSCalibur flow cytometer or were lysed with whole-cell extract buffer (50 mM Hepes, pH 7.4, 250 mM NaCl, 1% Nonidet 40, 1 mM EDTA) with 1 mM dithiothreitol (DTT), 1 mM phenylmethylsulfonyl fluoride (PMSF), and 1X protease inhibitor cocktail V (Calbiochem) for western analysis.

### IL-2 Intracellular Staining and Flow Cytometric Analysis

For stimulation, Jurkat and 5A8 cells were added with Brefeldin A (a Golgi transport inhibitor) to 24-well plates containing plate-bound anti-CD3 and soluble anti-CD28 antibodies for 16 h. Cells were collected by centrifugation, fixed with intracellular fixation buffer at room temperature for 20 min, washed, and stained with allophycocyanin-conjugated anti-human IL-2 in permeabilization buffer for 20 min. The cells were washed, resuspended in flow cytometry staining buffer, and analyzed with a FACSCalibur. All fixation and staining reagents were from eBioscience.

### Preparing Nuclear and Cytoplasmic Extracts

5A8 cells were treated with 500 nM CsA for 60 min and stimulated with phorbol-12-myristate-13-acetate [27] (20 nM) and ionomycin (2 µM) for various times. Nuclear and cytoplasmic extracts were obtained using the NE-PER nuclear and cytoplasmic extraction kit (Thermo Scientific).

### Immunoblot Analysis

Protein extracts in whole-cell, cytoplasmic, and nuclear extracts were quantified with a BCA assay (Thermo Scientific), separated by SDS-PAGE and transferred to a polyvinylidene difluoride membrane. The following western antibodies are employed: anti-IκBα (sc-203), anti-RelA (sc-372), anti-NFATc1 (sc-7294), anti-NFATc2 (sc-7296) (all from Santa Cruz Biotechnology), anti-NFATc3 (Cat. No. 4998, Cell Signaling Technology), anti-NFAT5 (PAI-023, Affinity BioReagent), anti-HDAC1 (a kind gift from E. Verdin, Gladstone Institutes), and monoclonal anti-β-actin (A5316, Sigma-Aldrich).

### In Vitro Kinase Assay

5A8 cells were incubated with 500 nM CsA for 60 min and stimulated with 20 nM PMA and 2 µM ionomycin for various times. At each time point, 5 million cells were lysed in whole-cell extract lysis buffer (see above) supplemented with 1 mM DTT, 1 mM PMSF, 10 mM *p*-nitrophenyl phosphate, 10 mM β-glycerophosphate, 200 µM sodium vanadate, 10 µg/ml aprotinin, and 1 µg/ml pepstatin and immunoprecipitated with antibodies against IκB kinase (IKK)-α(sc-7218, Santa Cruz Biotechnology). *In vitro* kinase assays using glutathione S-transferase IκBα (1–62) as the substrate were performed as described [10].

### Chromatin Immunoprecipitation Assay

5A8 cells were treated with DMSO or 500 nM CsA and stimulated with 200nM prostratin in the presence or absence of 2 µM ionomycin. Chromatin immunoprecipitation assays were performed as described [10] with modifications, specifically using protein A Dynabeads for antibody pulldown (Invitrogen) and 10% Chelex-100 (BioRad) for DNA elution [33]. The following antibodies were used: anti-RelA polyclonal antibody (sc-109) and rabbit control (both from Santa Cruz Biotechnology). Eluted immunoprecipitated DNA samples and corresponding input DNA at each time point were subjected to quantitative PCR with the 7900HT Sequence Detection System (Applied Biosystems), 2X QuantiTect probe PCR master mix (Qiagen), LTR-specific forward primer 5′-TTGACAGCCGCCTAGCATT-3′, reverse primer 5′-TTCTTGAAGTACTCCGGATGCA-3′, and Taqman probe 5′-CATCACATGGCCCGAGA-3′ designed with Primer Express software v.3.0 (Applied Biosystems). Enrichment was expressed as a percentage relative to input DNA.

### Establishing HIV Latency Model with Primary CD4 T Cells and Stimulation Conditions

Peripheral blood mononuclear cells (PBMC) were isolated by Ficoll-Hypaque density gradient centrifugation of buffy coats from HIV-seronegative donors (Stanford University Medical Center Blood Bank). Total CD4 T cells were isolated by negative selection with the EasySep CD4+ T-cell Enrichment Kit (Stem Cell Technologies). Isolated CD4 T cells were counted, collected as pellets by centrifugation at 200×*g* for 10 min at room temperature, and resuspended in the appropriate volume of concentrated viral supernatant. The use of a replication competent HIV-luciferase reporter virus for establishment of the latency model has been described previously [22], [23]. Typically, 800 ng of p24_Gag_ was used to spinoculate 1×10^7^ CD4 T cells in 15-ml Falcon conical tubes in volumes of 200 µl or less. Cells and virus were centrifuged at 1200×*g* for 1.5–2 h at room temperature. After spinoculation, cells were pooled and cultured at a concentration of 1×10^6^ cells/ml in RPMI 1640 with 5 µM saquinavir (Santa Cruz Biotechnology) for 3 days.

Cells were pretreated with or without 2 µM CsA, 30 µM rottlerin (Calbiochem) or 5 µM IKK-V (Calbiochem) for 60 min before they were stimulated by 1.5 µM ionomycin and various dosages of prostratin or human T-Activator CD3/CD28 (Invitrogen) Dynabeads for times as indicated. During this stimulation period, the culture medium contains 30 µM raltegravir (Santa Cruz Biotechnology) to ensure that the detected luciferase reporter activities came only from integrated proviruses.

## Results

### Characterization of a J-Lat Clone Inducible by T-cell Activation

**Figure 1:**
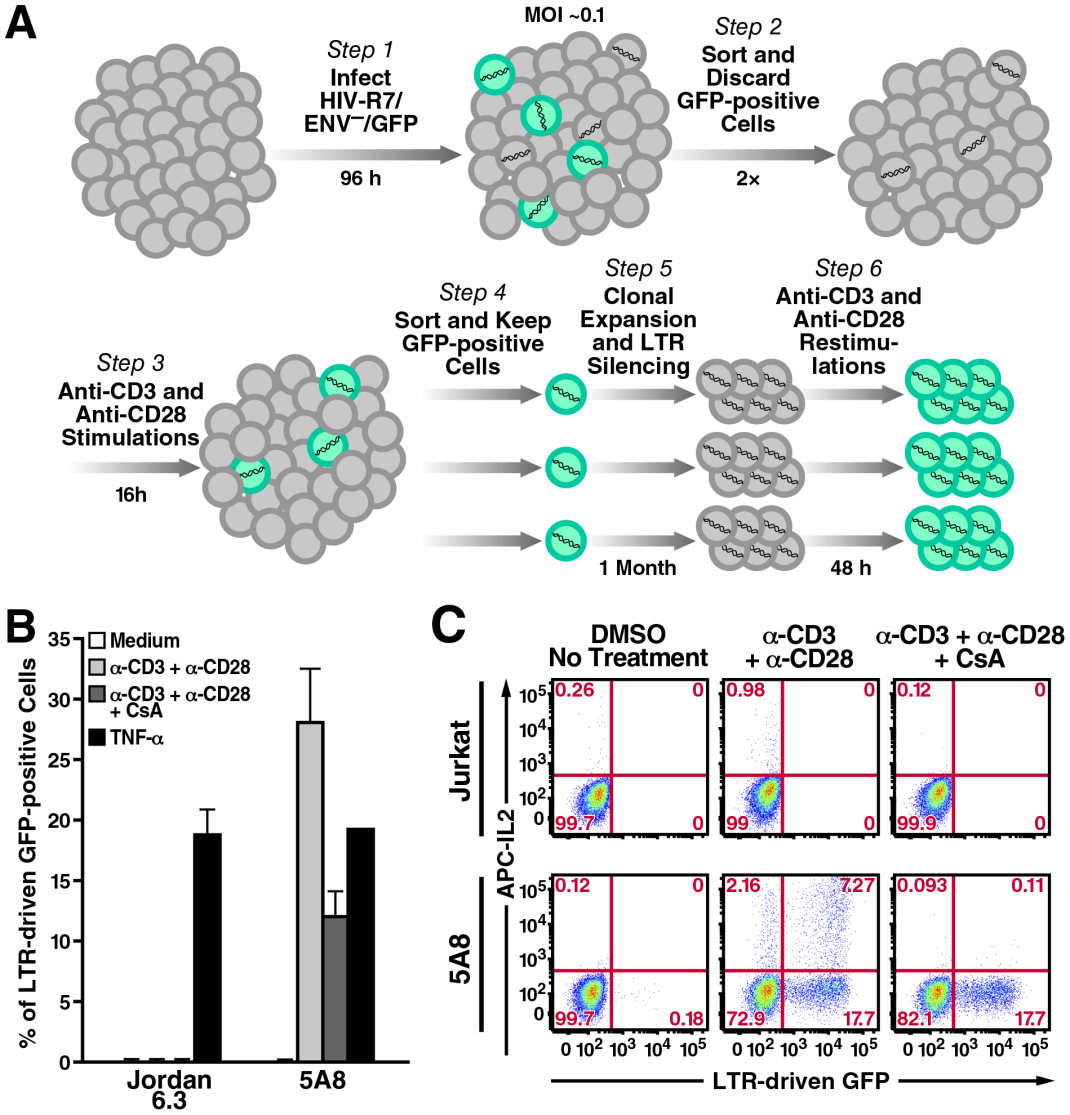
5A8 cells, a J-Lat cell line responsive to TCR stimulation and CsA inhibition. *A,* generation of 5A8 cells. *B,* LTR-GFP reporter activity of Jordan J-Lat 6.3 and 5A8 cells in response to different stimuli in the presence or absence of CsA. Cells were treated with DMSO (*white, light gray, black*) or 500 nM CsA (*dark gray*) and then left untreated *(white)* or stimulated with platebound anti-CD3 (10 µg/ml) and soluble anti-CD28 antibodies (2 µg/ml) (*light and dark gray*) or 10 ng/ml TNF-α (*black*) for 24 h, followed by FACS analysis to determine the percentage of GFP-positive cells (*y-axis*). Values are mean ± SEM from three experiments. Crosslinking of TCR induced latent HIV reporter activity in 5A8 cells that was partially inhibited by CsA. These responses were not observed in Jordan J-Lat 6.3 cells. *C,* TCR-crosslinking-dependent induction of IL-2 in Jurkat and 5A8 cells. Cells were treated with DMSO (*left and middle columns*) or 500 nM CsA (*right column*) and incubated with Brefeldin A alone or together with anti-CD3 and anti-CD28 antibodies prepared as above for 16 h. Cells were collected, stained with anti-human IL-2 antibody conjugated to allophycocyanin (APC), and analyzed by FACS. 5A8 cells produced more IL-2 than their parental Jurkat cells, probably because they were selected by their enriched expression of TCR complexes. CsA abolished IL-2 production but only partially inhibited LTR-driven GFP reporter activity.

Preliminary experiments revealed that the J-Lat HIV latency clones developed by Jordan and colleagues [30] are deficient in expression of the TCR subunits CD3 and CD28 (data not shown). To investigate the potential role of NFAT and NF-κB transcription family members in antagonizing HIV latency during T-cell activation, we employed the J-Lat clone 5A8, which was recently developed using TCR agonistic anti-CD3 and anti-CD28 antibodies as the selection stimuli (Fig. 1a) [34]. 5A8 harbors a full-length HIV-1 provirus that encodes *gfp* in lieu of *nef* as a reporter for HIV transcription. Integration site analysis revealed that this provirus is positioned in the sense intronic sequence of the adenosylmethionine transferase 2a gene (*MAT2a*). Similar to the Jordan J-Lat clones, 5A8 can display LTR driven-GFP reporter activity after TNF-α stimulation (Fig. 1b). Furthermore, upon TCR crosslinking, 5A8 undergoes HIV transcription and induces expression of NFAT-dependent cytokines, such as interleukin (IL)-2, in a CsA-sensitive manner (Fig. 1c). These findings argue that the calcium-signaling pathway linking TCR crosslinking and calcineurin activation in 5A8 cells is functionally intact, which allow us to analyze in detail the downstream effects of calcineurin on NFAT and NF-κB induction and in latent HIV-1 transcription.

### NFATs do not Participate in Latent HIV Transcription in 5A8 Cells

We first investigated which NFAT members mediate transcription of latent HIV-1. We focused on NFATc1, NFATc2, NFATc3, and NFAT5 because they are expressed in T cells, while NFATc4 is not. In the absence of stimulation, NFATc1, NFATc2, and NFATc3 are cytoplasmic. They are directly stimulated by calcium-activated calcineurin by dephosphorylation, which then translocate into the nucleus to mediate transcription. In fact, NFATc1 targets its own promoter, which leads to autoamplification of expression. NFAT5 is constitutively expressed in the nucleus. Its expression increases during T-cell activation by anti-CD3 crosslinking antibodies in a CsA-sensitive manner [35].

**Figure 2:**
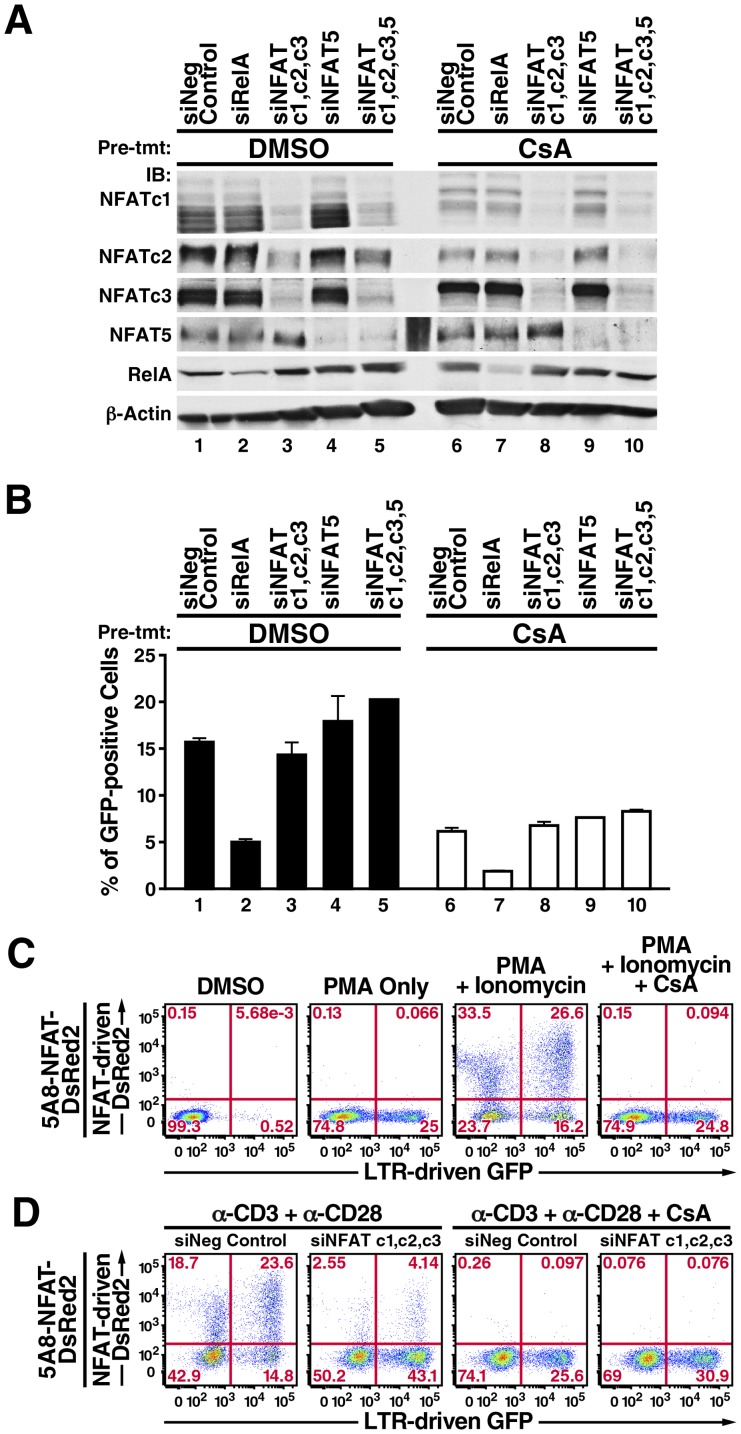
NFAT does not appear to be involved in latent HIV transcription in 5A8 cells. *A,* immunoblotting analysis of RelA and NFAT knockdowns in 5A8 cells. Negative control siRNA (lanes 1 and 6), siRNAs against RelA (lanes 2 and 7), NFATc1, NFATc2, and NFATc3 (lanes 3 and 8), NFAT5 alone (lanes 4 and 9), and all four NFATs (lanes 5 and 10) were introduced into 5A8 cells by Amaxa nucleofection twice within 48 h. Cells were then treated with DMSO (lanes 1 to 5) or 500 nM CsA (lanes 6 to 10) for 2 h, stimulated with anti-CD3 and anti-CD28 antibodies for 24 h, and collected for immunoblotting or FACS analyses. CsA reduced the levels of NFATc1 and NFATc2, which autoamplified their expressions after TCR crosslinking. *B,* FACS analysis of LTR-driven GFP reporter activity (*y-axis*). Values are mean ± SEM from four experiments. Reporter activity remained partially sensitive to CsA inhibition (lanes 6 to 10) but was insensitive to NFAT knockdown (lanes 3 to 5). *C,* characterization of 5A8-NFAT-DsRed2 cells. Cells were pretreated with DMSO (*panels 1–3*) or 500 nM CsA for 2 h (*panel 4*) and incubated with 20 nM PMA (*panel 2*), or 20 nM PMA and 2 µM ionomycin (*panels 3 and 4*) for 24 h, followed by FACS analysis. PMA alone induced activity of the GFP reporter but not the DsRed2 reporter (*panel 2*). Ionomycin and PMA induced both reporters (*panel 3*). CsA fully inhibited combined PMA/ionomycin induction of DsRed2 reporter but only partially suppressed GFP reporter activity to levels similar to those after stimulation with PMA only (*panel 4*). *D,* effects of NFATc1, NFATc2 and NFATc3 knockdown on expression of NFAT-dependent DsRed2 and LTR-driven GFP. 5A8-NFAT-DsRed2 cells were nucleofected with negative control siRNA (*panels 1 and 3*) or siRNA against NFATc1, NFATc2, and NFATc3 (*panels 2 and 4*) twice within 48 h, treated with DMSO (*panels 1 and 2*) or 500 nM CsA (*panels 3 and 4*) for 2 h, and stimulated with anti-CD3 and anti-CD28 antibodies as in Figure 1. NFATc1, NFATc2 and NFATc3 knockdown decreased expression of the DsRed2 reporter but did not inhibit GFP reporter activity (*panels 1 and 2*). CsA partially reduced GFP reporter expression even in the absence of NFAT (*panels 2 and 4*).

Since these factors are potentially redundant in transcriptional specificities, we introduced into 5A8 cells single or multiple siRNAs against all three calcineurin-activated NFATs (Fig. 2a, lanes 3 and 8), against NFAT5 alone (Fig. 2a, lanes 4 and 9), or against all four NFATs (Fig. 2a, lanes 5 and 10). The cells were pretreated with CsA (or left untreated) and stimulated with anti-CD3 and anti-CD28 antibodies. As expected, CsA substantially suppressed the autoamplification of NFATc1 and lowered NFATc2 expression (Fig. 2a, compare lanes 1, 2, and 4 with 6, 7, and 9), but did not suppress expression of NFAT5 (Fig. 2a, compare lanes 1–3 with 6–8). This suggests that unlike TCR crosslinking by anti-CD3 antibody alone [35], crosslinking both anti-CD3 and anti-CD28 can sustain NFAT5 expression in a calcineurin-independent manner.

We next compared HIV transcription levels under different NFAT-knockdown conditions to those in cells expressing siRNA negative control (Fig. 2a, lanes 1 and 6) or siRNA against NF-κB/RelA knockdown (Fig. 2a, lanes 2 and 7). Unexpectedly, reduction of NFATc1, NFATc2, NFATc3, and/or NFAT5 levels did not compromise HIV-1 transcription (Fig. 2b, compare lanes 1 and 3–5) or add to the effect of CsA-mediated inhibition of latent HIV transcription (Fig. 2b, compare lanes 6 and 8–10). However, NF-κB/RelA knockdown substantially reduced latent HIV transcription (Fig. 2b, compare lanes 1 and 2), and this inhibitory effect was enhanced by CsA (Fig. 2b, compare lanes 6 and 7). These results confirm that latent HIV transcription is CsA-sensitive (Fig. 1b) but suggest that it does not involve NFAT action.

Could NFAT factors that persist after knockdown have mediated the observed level of latent HIV transcription? To answer this question, we engineered 5A8 cells to contain a stable DsRed2 reporter regulated by a synthetic promoter composed of four NFAT-binding consensus sequences (5A8-NFAT-DsRed2 cells). NFATs induced by calcium signaling alone have weak activity [36]. Full activity requires transcription factors that have a strong transactivation domain. One prominent partner is the heterodimeric AP-1 complex consisting of c-fos and c-jun, which in turn is activated upon TCR crosslinking via a protein kinase C (PKC)-Ras-mitogen-activated protein kinase pathway [37]. In 5A8-NFAT-DsRed2 cells, PKC agonists such as PMA were insufficient to drive expression of the DsRed2 reporter (Fig. 2c, panel 2). However, DsRed2 reporter activity was robustly induced by PMA and ionomycin (Fig. 2c, panel 3). This effect was abolished by CsA (Fig. 2c, panel 4).

We then assessed the combined effect of NFATc1, NFATc2, and NFATc3 knockdown on DsRed2 and GFP reporter activities upon TCR crosslinking. NFAT-dependent DsRed2 reporter activity was greatly diminished, but LTR-dependent GFP reporter activity was unaltered (Fig. 2d, panel 2). After treatment with CsA, DsRed2 reporter activity was abolished, but LTR reporter activity was partially inhibited (Fig. 2d, panel 3). Knockdown of NFATc1, NFATc2, and NFATc3 did not increase the inhibition (Fig. 2d, panel 4). We conclude that NFATs can induce genes such as IL-2 but are unlikely to be the calcium-responsive factors that drive latent HIV transcription in 5A8 cells.

### Crosstalk of Calcium/Calcineurin Signaling Augments NF-κB-driven Latent HIV Transcription in 5A8 Cells

The prototypical NF-κB RelA/p50 complex is normally sequestered in the cytoplasm by IκBα proteins. TCR crosslinking or stimulation with phorbol esters induces PKC to associate with the Carma-1-Bcl-10-Malt-1 (CBM) complex, which together activates IKK signalosome to phosphorylate the IκB proteins. This leads to IκBα ubiquitination and degradation by the 26S proteasome, releasing NF-κB RelA/p50 into the nucleus where it exerts its transactivation function [27], [29], [38].

**Figure 3:**
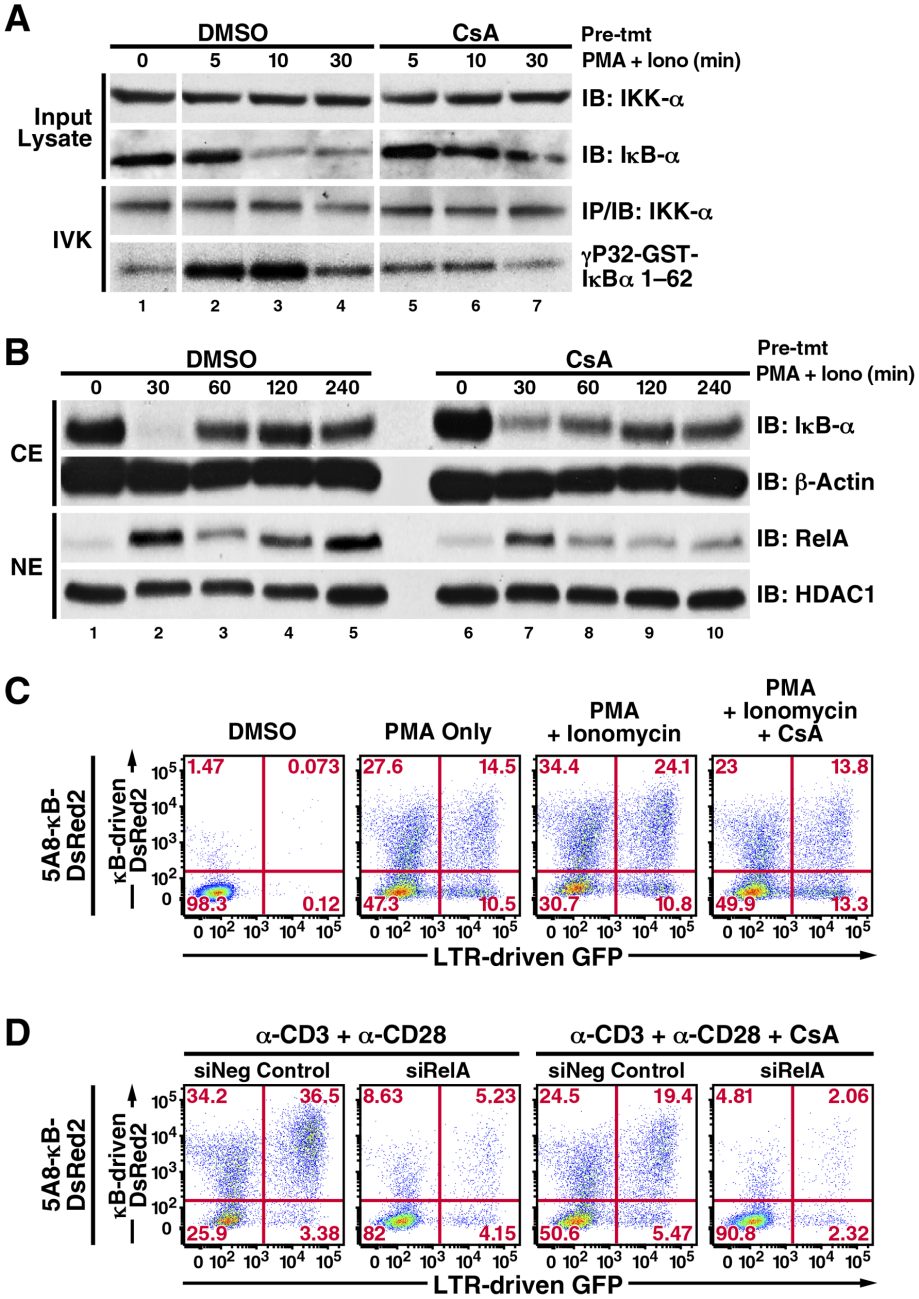
CsA reduces PKC-induced NF-κB/RelA activation and LTR-transcription in 5A8 cells. *A, in vitro* kinase assay of IκBα phosphorylation by IKK. 5A8 cells were pretreated with DMSO or 500 nM CsA for 2 h, stimulated with 20 nM PMA and 2 µM ionomycin for the indicated times, lysed, and *in vitro* kinase assays was performed using glutathione-S-transferase IκBα (1–62) as the substrate as described [10]. CsA delayed the degradation of endogenous IκBα induced by PMA/ionomycin, which correlated with reduced phosphorylation of GST-IκBα by immunoprecipitated IKK-α. *B*, analysis of PMA/ionomycin-induced IκBα degradation and RelA nuclear translocation. 5A8 cells were treated with DMSO or 500 nM CsA for 2 h, stimulated with 20 nM PMA and 2 µM ionomycin, and fractionated into nuclear and cytoplasmic extracts. Immunoblotting analyses revealed that CsA interfered with complete degradation of cytoplasmic IκBα at 30 min and reduced its reappearance at 60 min. CsA also reduced the first and second rounds of nuclear NF-κB/RelA expression at 30 min and 120 min. *C,* characterization of 5A8-κB-DsRed2 cells. Cells were treated as in Figure 2C. Unlike 5A8-NFAT-DsRed2 cells, PMA alone induced both κB-dependent DsRed2 and LTR-driven GFP reporter activities (*panel 2*). Combined PMA/ionomycin stimulation further enhanced the activities of both reporters (*panel 3*), which were partially suppressed by CsA to levels similar to those after stimulation with PMA only (*panel 4*). *D,* effects of RelA knockdown on expression of κB-dependent DsRed2 and LTR-driven GFP. 5A8-κB-DsRed2 cells were nucleofected with negative control siRNA (*panels 1 and 3*) or siRNA against RelA (*panels 2 and 4*) twice within 48 h, followed by drug and antibody treatments as in Figure 2d. RelA knockdown suppressed both κB-DsRed2 and LTR-driven GFP reporter activities (*panels 2 and 4*).

Recently, calcium/calcineurin signaling was shown to be important for NF-κB activation in T cells by stabilizing the assembly of the CBM complex [27]. In agreement to this work, CsA reduced the levels of PMA/ionomycin induced IκBα phosphorylation and degradation, and RelA nuclear translocation in 5A8 cells (Fig. 3a, b). We also introduced a stable κB-DsRed2 reporter controlled by tandem NF-κB consensus sequences into 5A8 cells (5A8-κB-DsRed2 cells) and observed that, after TCR crosslinking or PMA/ionomycin stimulations, both DsRed2 and GFP reporter activities were sensitive to CsA inhibition (Fig. 3c, panel 4; Fig. 3d panels 3, 4). Importantly, activities of both reporters were abolished upon RelA-knockdown by siRNA (Fig. 3d, panels 2, 4), supporting the idea that calcium/calcineurin signaling enhances the induction of NF-κB/RelA to target these reporters.

**Figure 4:**
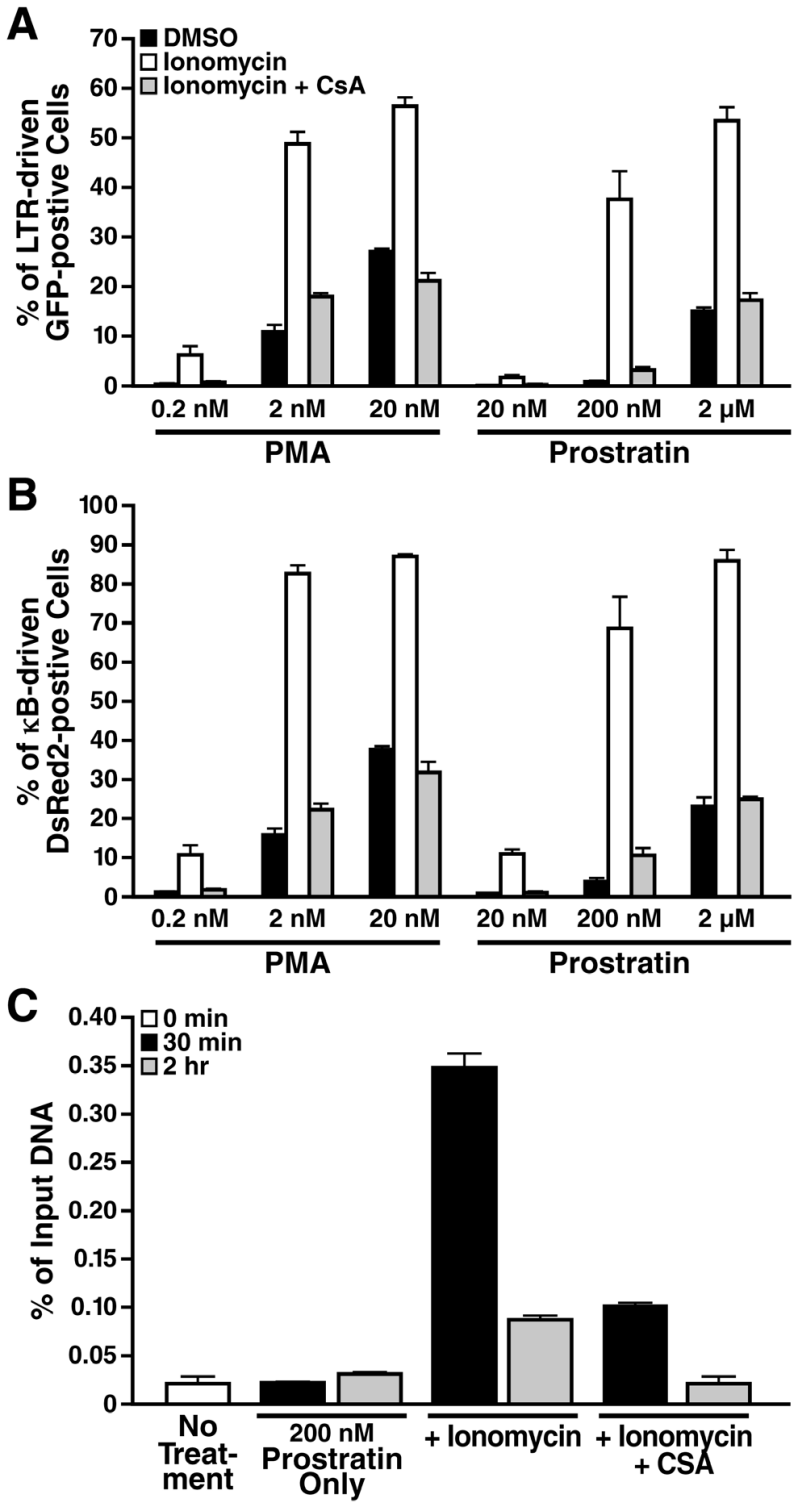
Calcium/calcineurin signaling synergizes with prostratin to antagonize latent HIV-1 by targeting RelA to LTR. *A and B,* dose-response analyses of LTR-driven GFP and κB-driven DsRed2 reporter activities. 5A8 cells were pretreated with DMSO (black and white bars) or 500 nM CsA (gray bars) for 2 h, followed by stimulation with PMA or prostratin only (black bars), or together with 2 µM ionomycin (white and gray bars) for 24 h at the concentration of the phorbol ester as indicated before FACS analysis. Values are mean ± standard deviation from one representative experiment. CsA potently suppressed the synergistic effect of ionomycin with PMA or prostratin and reverted back the activities of DsRed2 and GFP reporters to PMA- or prostratin-only levels. *C,* ChIP analysis of RelA recruitment to HIV-1 LTR. 5A8 cells were pretreated with DMSO or 500 nM CsA, followed by stimulation with 200 nM prostratin alone or together with 2 µM ionomycin for times indicated. Cells were fixed and subjected to chromatin immunoprecipitation with anti-RelA antibodies. Enrichment of LTR-chromatin in anti-RelA immunoprecipitates was expressed as a percentage of input chromatin. Ionomycin synergized with suboptimal dosage of prostratin to promote robust to substantial RelA occupancy at HIV-1 LTR at 30 min and 2 h, respectively. CsA effectively diminished RelA recruitment.

We further investigated whether ionomycin synergizes with PKC agonists, such as prostratin, which is a naturally occurring non-tumorigenic phorbol ester. We found that ionomycin synergized with prostratin as effectively as with PMA in activating both NF-κB-dependent DsRed2 and LTR-dependent GFP reporters. Such powerful synergism was observed even when prostratin was used at suboptimal dosages such as 200 nM, which by itself was insufficient to induce any robust IκBα degradation or reporter activities (Fig. 4a, b and data not shown). In all prostratin dosages tested, CsA consistently counteracted their synergistic effect with ionomycin. To confirm that calcium/calcineurin signaling affects NF-κB/RelA occupancy at HIV-LTR, we performed chromatin immunoprecipitation analysis in 5A8 cells stimulated with prostratin in the presence or absence of ionomycin and CsA. We found that ionomycin robustly synergized with suboptimal level of prostratin to promote NF-κB/RelA recruitment at HIV-LTR shortly after stimulation (Fig. 4c, 30 min). This recruitment remained substantial at a later time point (Fig. 4c, 2 h). In contrast, NF-κB/RelA recruitment was not robust but quickly declined to the baseline level in cells pretreated with CsA (Fig. 4c).

These experiments suggest that calcium/calcineurin signaling synergizes with the PKC-CBM-IKK pathway to allow maximal and sustained nuclear translocation and transactivation by RelA at the HIV LTR.

### Inhibiting PKC or IKK Abolishes the Synergistic Effect of Calcineurin and Prostratin on Latent HIV Transcription in Primary CD4+ T Cells

Finally, we proceeded to confirm that the synergistic effect between calcium/calcineurin signaling and prostratin to induce NF-κB dependent HIV transcription is conserved in a latency model we recently published [22]. This model involves a spinoculation step to infect resting primary CD4 T cells purified from blood PBMCs, using HIV viruses which harbor a luciferase reporter gene in lieu of *nef*. Spinoculation enables a high degree of cellular attachment to viruses, which in turn leads to a substantial portion of the infecting viruses that can complete reverse transcription, integration and establishment of transcriptional latency in the infected cells within 72 h [23]. Here, we pretreated spinoculated cells with various calcineurin inhibitors (CsA and FK-506) or kinase inhibitors (IKK-2 inhibitor V and PKC inhibitor rottlerin), followed by their stimulation with cellular or pharmacological agents for 30 h before we assessed their HIV reporter activities. To ensure that the luciferase activities came exclusively from post-integrated proviruses, we added the potent integrase inhibitor, raltegravir, into the culture medium during stimulation.

**Figure 5:**
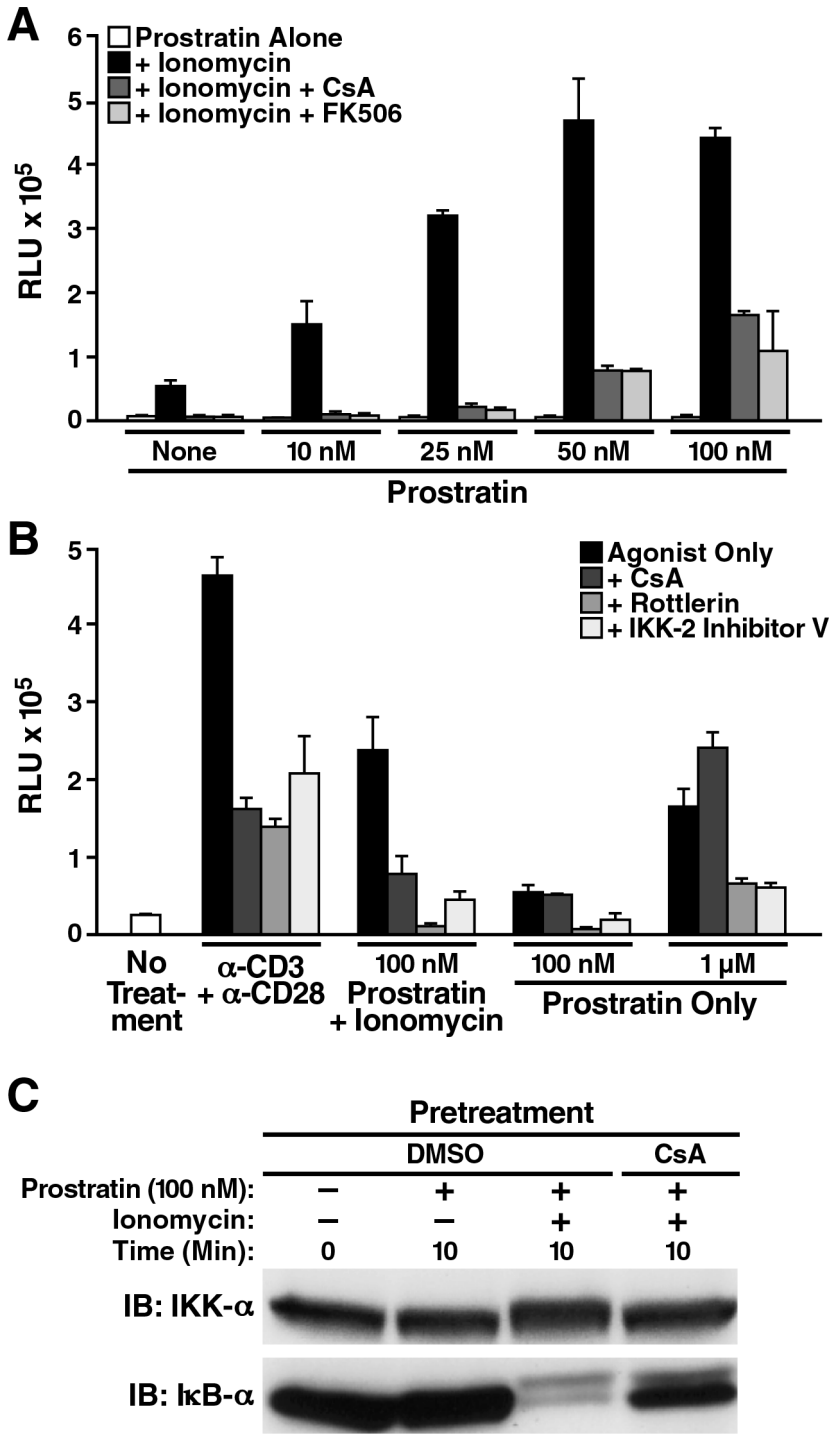
PKC/IKK inhibition strongly reduces prostratin/ionomycin-induced latent HIV transcription in primary CD4+ T cells. *A* and *B*, evaluating inhibitors of relevant pathway in suppressing NL4-3 luciferase reporter activities in latently infected primary CD4+ T cells. *A,* latently infected cells pretreated with DMSO (white and black bars), calcineurin inhibitors CsA (dark gray bar) or FK-506 (light gray bar) were incubated in medium that contained no or suboptimal dosages of prostratin (0 to 100 nM, white bars), or contained both prostratin and 1.5 µM ionomycin (black, dark and light gray bars) for 48 h. Either calcineurin inhibitors inhibited the dose-dependent synergistic effect between suboptimal dosages of prostratin and ionomycin on luciferase reporter activities. *B,* latently infected cells pretreated with DMSO (white and black bars) or various kinase/phosphatase inhibitors (dark, medium and light gray bars) were incubated in media alone (white bar), or media containing anti-CD3/anti-CD28 Dynabeads (1∶1 ratio), or prostratin, in the presence or absence of 1.5 µM ionomycin as indicated for 30 h. All inhibitors suppressed reporter activities induced by TCR crosslinking or prostratin/ionomycin, which involved induction of calcium/calcineurin signaling, but CsA was ineffective against reporter activities induced by prostratin alone. In both *A* and *B*, the RLU were normalized based on total protein present in the various cell lysates. All stimulations were performed in triplicate with error bars representing ± standard deviation. Results are representative of experiments performed with cells from three independent donors. *C*, analysis of prostratin/ionomycin-induced IκBα degradation in primary CD4 cells. PBMC-purified CD4 cells were treated with DMSO or 500 nM CsA for 2 h, stimulated with a suboptimal dose of prostratin at 100 nM in the presence or absence of 1.5 µM ionomycin for 10 min and whole-cell lysate was prepared. Immunoblotting analyses revealed that CsA effectively reduced the effect of stimulus-coupled degradation of cytoplasmic IκBα.

We found that ionomycin indeed synergizes with suboptimal dosages of prostratin to induce HIV reporter activity to levels similar to that achieved by TCR crosslinking (Figures 5a and 5b). Notably, calcineurin inhibitors are interchangeably as effective as rottlerin and IKK-2 inhibitor V in suppressing HIV reporter activity (Figures 5a, b), and in curtailing the degradation of IκBα (Figures 5c). In contrast, CsA cannot inhibit HIV reporter activity stimulated by prostratin alone (Figure 5b). We also attempted to introduce siRNA/shRNAs against the NFATs into the spinoculated cells. However, this led to rapid cellular toxicity, precluding further detailed analysis (data not shown). We therefore conclude that in this primary CD4 T-cell model of HIV latency, NF-κB is most likely the predominant downstream target of the synergistic signaling between calcium/calcineurin and PKC to facilitate robust HIV transcription.

## Discussion

In this study, we found no compelling evidence that NFAT contributes to the transcriptional activation of latent HIV in a J-Lat model we recently developed that robustly displays TCR-induced calcium/calcineurin signaling (Figs. 1 and 2). Prior studies showed that NFATc1, NFATc2, and NFAT5 are important positive regulators of HIV transcription during acute infection in T cells and macrophages [17]–[20]. Some of these differences might simply reflect distinct functions of NFAT members in various cellular settings. For example, NFAT5 might promote HIV transcription in macrophages but not in T cells. Since NFATc1 and NFATc2 bind to the LTR *in vitro* and enhance HIV infection in T-cell lines and primary CD4 cells [17]–[19], it is intriguing that they did not contribute to latent HIV transcription in 5A8 cells.

In most previous studies, select NFAT members or the HIV reporter were overexpressed or their endogenous levels were increased by prolonged cellular or mitogen stimulation (sometimes for days). The resulting NFAT levels are likely higher than those in unstimulated 5A8 cells or in 5A8 cells induced by TCR crosslinking for a maximum of 24 h. Importantly, in biophysical studies, a p50/RelA heterodimer associated with HIV LTR κB site tightly with a K_d_, 0.1 nM to 0.1 pM, whereas a NFATc2 dimer bound with much less avidity (K_d_, 20 nM). Assuming equal cellular concentrations of NFAT and p50/RelA in latently infected resting CD4 cells, NFAT would compete poorly with p50/RelA for binding to the LTR. p50/RelA is abundant under basal conditions and can be mobilized to the nucleus within minutes after stimulation (Fig. 3). Nonetheless, we speculate that in CD4 T cells that are acutely infected with HIV and immune activated for days, NFAT could be expressed at a higher level than RelA. If so, NFAT might possibly exert a cooperative or dominant effect over RelA on HIV transcript production.

Our data support observations in Jurkat cells and resting primary CD4 T cells that the CsA-sensitive calcineurin augments the PKC-dependent induction of NF-κB/RelA to target the latent HIV-1 LTR (Figs. 3, 4, 5). The underlying mechanism is elegantly shown by Palkowitsch and colleagues (2011) and involves the dephosphorylating action of calcineurin on Bcl-10 at its carboxyl serine rich region (residues 130–147), which by default is phosphorylated by an unknown kinase. Unphosphorylated Bcl-10 efficiently assembles with Carma-1 and Malt-1 to form the CBM complex, which links PKC and the IKK signalosome, resulting in robust activation of NF-κB [27]. Our study is the first to demonstrate that this calcium/calcineurin signaling crosstalk is conserved, using prostratin as the PKC agonist (Fig. 4). Likewise, the Grundström group [39] has recently shown that in addition to its stimulatory effect on calcineurin, ionomycin activates Calcium/calmodulin dependent kinase II (CaMKII) to phosphorylate Bcl-10 at threonine 91. This modification triggers Bcl-10 and bound IKKγ to undergo K63-linked polyubiquitination, which in turn is essential for robust activation of the IKK signalosome and NF-κB/RelA. Together, our result and theirs have highlighted an unexpected role of classical calcium-responsive pathways (CaMK and calcineurin) in inducing the activation of NF-κB in T cells. We speculate that other calcium-responsive pathways targeting NF-κB binding to HIV LTR might also exist. For example, the Cron group [40] has recently shown that the transcription factor c-Maf binds to a Maf response element (MARE) immediate upstream of the LTR κB sites and synergizes with NF-κB/RelA and NFATc1 to drive HIV transcription during acute infection. Currently, the role of c-Maf in latent HIV transcription is unclear. For future studies, it might be of interest to investigate whether calcium-responsive pathways induce the interaction between c-Maf and NF-κB/RelA, which in turn stabilizes and enhances NF-κB/RelA binding to the latent HIV LTR.

Due to its low cellular toxicity, non-tumorigenicity and ability to downregulate CD4 receptors to prevent unwarranted new rounds of infection, prostratin has been widely regarded as a promising “flushing” reagent for eradicating latent HIV-1 reservoir. However, prostratin is only extracted in low and variable amounts from natural sources; synthesizing it at an industrial scale with pharmaceutical grade material has yet to be achieved [41]. We would like to propose that identifying and developing inhibitors of the putative kinase that phosphorylates the carboxyl serine-rich region of Bcl-10 at the basal state could represent a powerful adjuvant strategy that boosts prostratin action. First, it curtails inhibitory phosphorylation on endogenous Bcl-10, such that the PKC-CBM-IKK supramolecular complex formation can occur using prostratin at a much lower dose. Moreover, it obviates activating calcineurin itself and undesirably its other downstream targets (e.g., NFATs). If proved valid, such strategy might promote effective purging of the latent reservoir without eliciting adverse immune effects associated with generalized T-cell activation.

Studies of primary T models of HIV latency have increased our understanding of the molecular underpinnings of HIV latency *in vivo*. Strikingly, the relationships between NFAT and NF-κB induction pathways and latent HIV transcription in each of these models are unique. For examples, in the Bosque latency model, various pharmacological inhibitors along the PKC-CBM-IKK-NF-κB pathway do not suppress HIV transcription induced by TCR crosslinking or phytohemagglutinin in cells with a central memory T-cell phenotype [21]. Rather, this process was blocked by CsA, arguing for an exclusive role for NFAT transcription factors in the anti-latency response. Indeed the authors concluded that reactivation of latent virus in these cells principally proceeds through a Lck-calcineurin-NFAT pathway. On the other hand, in the Yang latency model, phorbol ester or ionomycin can induce transcription of latent virus in CD4 cells transduced with anti-apoptotic factor Bcl-2, an anti-apoptotic factor that confers an effector memory T-cell phenotype. They concluded that NF-κB and NFATs are both involved in reactivating latent HIV-1. We speculate that the relative contributions NF-κB and NFAT in regulating viral persistence could be specific to distinct memory subsets, an intriguing possibility that merits further investigation. However, results from our J-Lat and primary latency models strongly provide a cautionary note that CsA inhibition does not necessarily equate with NFAT participation in latent HIV-1 transcription. Besides, phytohemagglutinin activates NF-κB in an EGTA-sensitive manner [26]. Detailed analysis with a tractable method to efficiently knockdown NFAT and NF-κB in various resting primary CD4+ cell populations is required to ultimately address this intriguing question.
